# 2-Phenoxy­acetohydrazide

**DOI:** 10.1107/S1600536809051344

**Published:** 2009-12-04

**Authors:** Hoong-Kun Fun, Ching Kheng Quah, Arun M. Isloor, Dhanya Sunil, Prakash Shetty

**Affiliations:** a X-ray Crystallography Unit, School of Physics, Universiti Sains Malaysia, 11800 USM, Penang, Malaysia; b Department of Chemistry, National Institute of Technology-Karnataka, Surathkal, Mangalore 575 025, India; c Department of Chemistry, Manipal Institute of Technology, Manipal University, 576 104, India; d Department of Printing and Media Engineering, Manipal Institute of Technology, Manipal University, 576 104, India

## Abstract

In the title compound, C_8_H_10_N_2_O_2_, the acetohydrazide group is almost planar, with an r.m.s. deviation of 0.028 Å. In the crystal, the mol­ecules are linked by inter­molecular C—H⋯O, N—H⋯O and N—H⋯N hydrogen bonds into infinite sheets lying parallel to (001). The acetohydrazide O atom accepts two N—H⋯O links and one C—H⋯O link.

## Related literature

For general background to and biological properties of hydrazine derivatives, see: Rando *et al.* (2008 ▶); Kumar *et al.* (2009 ▶); Kamal *et al.* (2007 ▶); Masunari & Tavares (2007 ▶); Rando *et al.* (2002 ▶). For a related structure, see: Fun *et al.* (2009 ▶). For the preparation, see: Holla & Udupa (1992 ▶). For the stability of the temperature controller used for the data collection, see: Cosier & Glazer (1986 ▶).
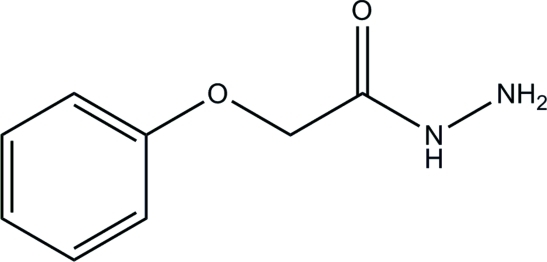


## Experimental

### 

#### Crystal data

C_8_H_10_N_2_O_2_
*M* *_r_* = 166.18
Monoclinic, 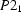

*a* = 6.3397 (8) Å
*b* = 4.0590 (6) Å
*c* = 15.948 (2) Å
β = 99.218 (10)°
*V* = 405.09 (10) Å^3^
*Z* = 2
Mo *K*α radiation
μ = 0.10 mm^−1^
*T* = 100 K
0.63 × 0.16 × 0.08 mm

#### Data collection

Bruker SMART APEXII CCD diffractometer
Absorption correction: multi-scan (*SADABS*; Bruker, 2005 ▶) *T*_min_ = 0.940, *T*_max_ = 0.992
3771 measured reflections
1063 independent reflections
916 reflections with *I* > 2σ(*I*)
*R*_int_ = 0.045

#### Refinement

*R*[*F* ^2^ > 2σ(*F* ^2^)] = 0.047
*wR*(*F* ^2^) = 0.124
*S* = 1.07
1063 reflections
121 parameters
1 restraint
H atoms treated by a mixture of independent and constrained refinement
Δρ_max_ = 0.25 e Å^−3^
Δρ_min_ = −0.25 e Å^−3^

### 

Data collection: *APEX2* (Bruker, 2005 ▶); cell refinement: *SAINT* (Bruker, 2005 ▶); data reduction: *SAINT*; program(s) used to solve structure: *SHELXTL* (Sheldrick, 2008 ▶); program(s) used to refine structure: *SHELXTL*; molecular graphics: *SHELXTL*; software used to prepare material for publication: *SHELXTL* and *PLATON* (Spek, 2009 ▶).

## Supplementary Material

Crystal structure: contains datablocks global, I. DOI: 10.1107/S1600536809051344/hb5256sup1.cif

Structure factors: contains datablocks I. DOI: 10.1107/S1600536809051344/hb5256Isup2.hkl

Additional supplementary materials:  crystallographic information; 3D view; checkCIF report

## Figures and Tables

**Table 1:** Hydrogen-bond geometry (Å, °)

*D*—H⋯*A*	*D*—H	H⋯*A*	*D*⋯*A*	*D*—H⋯*A*
N1—H1*N*1⋯N2^i^	0.86 (4)	2.21 (3)	2.953 (3)	144 (3)
N2—H1*N*2⋯O2^ii^	0.94 (4)	2.49 (3)	3.110 (3)	124 (2)
N2—H2*N*2⋯O2^iii^	0.96 (3)	2.05 (3)	2.986 (3)	163 (2)
C1—H1*A*⋯O2^iv^	0.93	2.51	3.396 (3)	159

Symmetry codes: (i) 
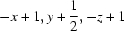
; (ii) 
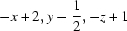
; (iii) 
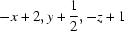
; (iv) 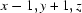.

**Fig. 1.**
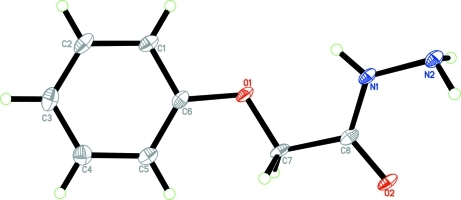
The molecular structure of (I), showing 30% probability displacement ellipsoids for non-H atoms.

**Fig. 2.**
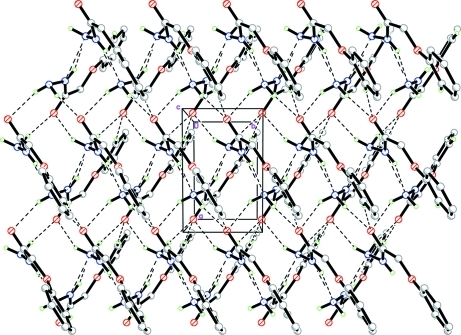
The crystal structure of (I) viewed along the c axis. H atoms not involved in intermolecular interactions (dashed lines) have been omitted for clarity.

## supplementary crystallographic information

### Comment

Hydrazine derivatives have been reported to possess several biological properties. 5-nitro-2-heterocyclic benzylidine hydrazides were found to possess antileishmanial activities (Rando *et al.*, 2008). Many substituted benzoic acid furan-2-yl-methylene hydrazides showed potent antimicrobial properties (Kumar *et al.*, 2009). Hydrazine derivatives were also associated with remarkable anticancer (Kamal *et al.*, 2007), antibacterial (Masunari & Tavares, 2007) and tuberculostatic (Rando *et al.*, 2002) activities.

The molecular structure is shown in Fig. 1. The acetohydrazide group (C7/C8/N1/N2/O2) is almost planar, with an r.m.s. deviation of 0.028 Å. Bond lengths and angles are within normal ranges, and comparable to a closely related structure (Fun *et al.*, 2009). In the crystal packing (Fig. 2), the molecules are linked *via* intermolecular C1—H1A···O2, N2—H1N2···O2 and N2—H2N2···O2 trifurcated acceptor bonds, together with N1—H1N1···N2 hydrogen bonds, into infinite two-dimensional networks parallel to plane (0 0 1).

### Experimental

Phenol (11 ml, 1.20 mmol), ethyl chloroacetate (12.8 ml, 1.20 mmol) and potassium carbonate (20.75 g, 1.50 mmol) were refluxed in acetone (100 ml) at 80 °C for 18 h. The reaction mixture was then filtered, distilled to remove the acetone and poured into ice cold water with vigorous stirring. The ester, phenoxy ethyl acetate was extracted using ether. The solution was distilled to remove ether. Phenoxy ethyl acetate (8.2 ml, 0.50 mmol) was heated at 100 °C for 10h in absolute alcohol medium (40 ml) with hydrazine hydrate (2.5 ml, 0.50 mmol). The reaction mixture was allowed to cool, the solid separated was filtered, dried and recrystallized from ethanol. The yield was found to be 5.7 g (69 %). *M. p.* 381-383 K (Holla & Udupa, 1992).

### Refinement

Atoms H1N1, H1N2 and H2N2 were located from the difference Fourier map and refined freely. The remaining H atoms were positioned geometrically and refined using a riding model, with C-H = 0.93 and 0.97 Å and *U*_iso_(H) = 1.2 *U*_eq_(C). In the absence of significant anomalous dispersion, 648 Friedel pairs were merged for the final refinement.

### Crystal data

**Table:**

C_8_H_10_N_2_O_2_	*F*(000) = 176
*M**_r_* = 166.18	*D*_x_ = 1.362 Mg m^−^^3^
Monoclinic, *P*2_1_	Mo *K*α radiation, λ = 0.71073 Å
Hall symbol: P 2yb	Cell parameters from 2286 reflections
*a* = 6.3397 (8) Å	θ = 3.3–30.1°
*b* = 4.0590 (6) Å	µ = 0.10 mm^−^^1^
*c* = 15.948 (2) Å	*T* = 100 K
β = 99.218 (10)°	Needle, colourless
*V* = 405.09 (10) Å^3^	0.63 × 0.16 × 0.08 mm
*Z* = 2	

### Data collection

**Table:**

Bruker SMART APEXII CCD diffractometer	1063 independent reflections
Radiation source: fine-focus sealed tube	916 reflections with *I* > 2σ(*I*)
graphite	*R*_int_ = 0.045
φ and ω scans	θ_max_ = 27.5°, θ_min_ = 2.6°
Absorption correction: multi-scan (*SADABS*; Bruker, 2005)	*h* = −8→8
*T*_min_ = 0.940, *T*_max_ = 0.992	*k* = −5→5
3771 measured reflections	*l* = −20→19

### Refinement

**Table:**

Refinement on *F*^2^	Primary atom site location: structure-invariant direct methods
Least-squares matrix: full	Secondary atom site location: difference Fourier map
*R*[*F*^2^ > 2σ(*F*^2^)] = 0.047	Hydrogen site location: inferred from neighbouring sites
*wR*(*F*^2^) = 0.124	H atoms treated by a mixture of independent and constrained refinement
*S* = 1.07	*w* = 1/[σ^2^(*F*_o_^2^) + (0.0857*P*)^2^] where *P* = (*F*_o_^2^ + 2*F*_c_^2^)/3
1063 reflections	(Δ/σ)_max_ < 0.001
121 parameters	Δρ_max_ = 0.25 e Å^−^^3^
1 restraint	Δρ_min_ = −0.24 e Å^−^^3^

### Special details

**Table:**

Experimental. The crystal was placed in the cold stream of an Oxford Cyrosystems Cobra open-flow nitrogen cryostat (Cosier & Glazer, 1986) operating at 100.0 (1) K.
Geometry. All esds (except the esd in the dihedral angle between two l.s. planes) are estimated using the full covariance matrix. The cell esds are taken into account individually in the estimation of esds in distances, angles and torsion angles; correlations between esds in cell parameters are only used when they are defined by crystal symmetry. An approximate (isotropic) treatment of cell esds is used for estimating esds involving l.s. planes.
Refinement. Refinement of F^2^ against ALL reflections. The weighted R-factor wR and goodness of fit S are based on F^2^, conventional R-factors R are based on F, with F set to zero for negative F^2^. The threshold expression of F^2^ > 2sigma(F^2^) is used only for calculating R-factors(gt) etc. and is not relevant to the choice of reflections for refinement. R-factors based on F^2^ are statistically about twice as large as those based on F, and R- factors based on ALL data will be even larger.

### Fractional atomic coordinates and isotropic or equivalent isotropic displacement parameters (Å^2^)

**Table:**

	*x*	*y*	*z*	*U*_iso_*/*U*_eq_	
O1	0.5379 (2)	0.5753 (5)	0.30334 (10)	0.0244 (5)	
O2	0.9591 (3)	0.0417 (5)	0.39131 (11)	0.0253 (5)	
N1	0.6740 (3)	0.2420 (6)	0.44403 (13)	0.0209 (5)	
N2	0.7205 (3)	0.0646 (7)	0.52119 (14)	0.0232 (5)	
C1	0.2408 (4)	0.8607 (7)	0.23319 (17)	0.0258 (6)	
H1A	0.1973	0.8907	0.2857	0.031*	
C2	0.1199 (4)	0.9836 (7)	0.16016 (18)	0.0303 (7)	
H2A	−0.0059	1.0965	0.1639	0.036*	
C3	0.1825 (4)	0.9417 (8)	0.08136 (18)	0.0309 (7)	
H3A	0.0997	1.0250	0.0326	0.037*	
C4	0.3696 (4)	0.7745 (8)	0.07658 (16)	0.0295 (7)	
H4A	0.4122	0.7443	0.0239	0.035*	
C5	0.4956 (4)	0.6502 (7)	0.14908 (16)	0.0258 (6)	
H5A	0.6228	0.5413	0.1453	0.031*	
C6	0.4291 (4)	0.6909 (7)	0.22719 (15)	0.0214 (6)	
C7	0.7239 (4)	0.3818 (7)	0.29959 (16)	0.0220 (6)	
H7A	0.8382	0.5223	0.2867	0.026*	
H7B	0.6931	0.2193	0.2547	0.026*	
C8	0.7937 (3)	0.2108 (7)	0.38313 (15)	0.0204 (5)	
H1N1	0.567 (5)	0.375 (11)	0.4326 (19)	0.041 (9)*	
H1N2	0.785 (5)	−0.132 (9)	0.5068 (17)	0.028 (8)*	
H2N2	0.822 (5)	0.192 (9)	0.5594 (16)	0.025 (8)*	

### Atomic displacement parameters (Å^2^)

**Table:**

	*U*^11^	*U*^22^	*U*^33^	*U*^12^	*U*^13^	*U*^23^
O1	0.0127 (8)	0.0297 (10)	0.0306 (9)	0.0070 (9)	0.0027 (6)	0.0003 (10)
O2	0.0106 (7)	0.0249 (10)	0.0406 (10)	0.0054 (9)	0.0043 (7)	−0.0005 (9)
N1	0.0087 (8)	0.0220 (12)	0.0313 (11)	0.0008 (9)	0.0011 (8)	0.0003 (10)
N2	0.0128 (9)	0.0240 (12)	0.0324 (12)	0.0001 (11)	0.0021 (8)	0.0002 (12)
C1	0.0160 (11)	0.0247 (15)	0.0372 (14)	0.0009 (12)	0.0056 (10)	0.0005 (13)
C2	0.0162 (11)	0.0268 (16)	0.0462 (16)	0.0008 (12)	0.0001 (11)	0.0014 (14)
C3	0.0267 (13)	0.0245 (14)	0.0373 (15)	−0.0017 (14)	−0.0072 (11)	0.0023 (13)
C4	0.0320 (14)	0.0251 (16)	0.0308 (13)	0.0006 (14)	0.0034 (11)	0.0004 (13)
C5	0.0192 (12)	0.0235 (15)	0.0348 (13)	0.0008 (12)	0.0043 (10)	−0.0014 (12)
C6	0.0143 (10)	0.0179 (12)	0.0311 (13)	−0.0030 (11)	0.0003 (9)	0.0004 (12)
C7	0.0098 (10)	0.0221 (14)	0.0345 (13)	0.0019 (11)	0.0048 (9)	−0.0033 (12)
C8	0.0102 (10)	0.0172 (11)	0.0329 (13)	−0.0029 (11)	0.0004 (9)	−0.0045 (12)

### Geometric parameters (Å, °)

**Table:**

O1—C6	1.379 (3)	C2—C3	1.388 (4)
O1—C7	1.425 (3)	C2—H2A	0.9300
O2—C8	1.243 (3)	C3—C4	1.379 (4)
N1—C8	1.331 (3)	C3—H3A	0.9300
N1—N2	1.416 (3)	C4—C5	1.391 (4)
N1—H1N1	0.86 (4)	C4—H4A	0.9300
N2—H1N2	0.94 (4)	C5—C6	1.387 (3)
N2—H2N2	0.96 (3)	C5—H5A	0.9300
C1—C2	1.381 (4)	C7—C8	1.505 (4)
C1—C6	1.395 (3)	C7—H7A	0.9700
C1—H1A	0.9300	C7—H7B	0.9700
			
C6—O1—C7	116.79 (18)	C3—C4—H4A	119.4
C8—N1—N2	121.5 (2)	C5—C4—H4A	119.4
C8—N1—H1N1	115 (2)	C6—C5—C4	119.1 (2)
N2—N1—H1N1	123 (2)	C6—C5—H5A	120.4
N1—N2—H1N2	104.9 (17)	C4—C5—H5A	120.4
N1—N2—H2N2	107.6 (19)	O1—C6—C5	124.7 (2)
H1N2—N2—H2N2	110 (3)	O1—C6—C1	114.9 (2)
C2—C1—C6	119.1 (2)	C5—C6—C1	120.5 (2)
C2—C1—H1A	120.5	O1—C7—C8	110.18 (19)
C6—C1—H1A	120.5	O1—C7—H7A	109.6
C1—C2—C3	121.2 (2)	C8—C7—H7A	109.6
C1—C2—H2A	119.4	O1—C7—H7B	109.6
C3—C2—H2A	119.4	C8—C7—H7B	109.6
C4—C3—C2	118.9 (2)	H7A—C7—H7B	108.1
C4—C3—H3A	120.5	O2—C8—N1	123.1 (2)
C2—C3—H3A	120.5	O2—C8—C7	118.1 (2)
C3—C4—C5	121.1 (2)	N1—C8—C7	118.7 (2)
			
C6—C1—C2—C3	−0.1 (4)	C2—C1—C6—O1	−179.5 (2)
C1—C2—C3—C4	−0.1 (4)	C2—C1—C6—C5	0.9 (4)
C2—C3—C4—C5	−0.4 (5)	C6—O1—C7—C8	−166.8 (2)
C3—C4—C5—C6	1.2 (4)	N2—N1—C8—O2	−4.2 (4)
C7—O1—C6—C5	−4.4 (4)	N2—N1—C8—C7	174.4 (2)
C7—O1—C6—C1	176.0 (2)	O1—C7—C8—O2	−177.3 (2)
C4—C5—C6—O1	179.0 (2)	O1—C7—C8—N1	4.0 (3)
C4—C5—C6—C1	−1.4 (4)		

### Hydrogen-bond geometry (Å, °)

**Table:**

*D*—H···*A*	*D*—H	H···*A*	*D*···*A*	*D*—H···*A*
N1—H1N1···N2^i^	0.86 (4)	2.21 (3)	2.953 (3)	144 (3)
N2—H1N2···O2^ii^	0.94 (4)	2.49 (3)	3.110 (3)	124 (2)
N2—H2N2···O2^iii^	0.96 (3)	2.05 (3)	2.986 (3)	163 (2)
C1—H1A···O2^iv^	0.93	2.51	3.396 (3)	159

Symmetry codes: (i) −*x*+1, *y*+1/2, −*z*+1; (ii) −*x*+2, *y*−1/2, −*z*+1; (iii) −*x*+2, *y*+1/2, −*z*+1; (iv) *x*−1, *y*+1, *z*.

## References

[bb1] Bruker (2005). *APEX2*, *SAINT* and *SADABS* Bruker AXS Inc., Madison, Wisconsin, USA.

[bb3] Cosier, J. & Glazer, A. M. (1986). *J. Appl. Cryst.* **19**, 105–107.

[bb4] Flack, H. D. (1983). *Acta Cryst.* A**39**, 876–881.

[bb5] Fun, H.-K., Quah, C. K., Sujith, K. V. & Kalluraya, B. (2009). *Acta Cryst.* E**65**, o1184–o1185. 10.1107/S1600536809015542 PMC2969560 21583058

[bb2] Holla, B. S. & Udupa, K. V. (1992). *Farmaco*, **47**, 305–318. 1503594

[bb6] Kamal, A., Khan, N. A., Reddy, K. S. & Rohini, K. (2007). *Bioorg. Med. Chem.* **15**, 1004–1013. 10.1016/j.bmc.2006.10.027 17097292

[bb7] Kumar, P., Narasimhan, B., Sharma, D., Judge, V. & Narang, R. (2009). *Eur. J. Med. Chem.* **44**, 1853–1863. 10.1016/j.ejmech.2008.10.034 19081655

[bb8] Masunari, A. & Tavares, L. C. (2007). *Bioorg. Med. Chem.* **15**, 4229–4236. 10.1016/j.bmc.2007.03.068 17419064

[bb9] Rando, D. G., Avery, M. A., Tekwani, B. L., Khan, S. I. & Ferreira, E. I. (2008). *Bioorg. Med. Chem.* **16**, 6724–6731. 10.1016/j.bmc.2008.05.076 18571927

[bb10] Rando, D. G., Sato, D. N., Siqueira, L., Malvezzi, A., Leite, C. Q. F., do Amaral, A. T., Ferreira, E. I. & Tavares, L. C. (2002). *Bioorg. Med. Chem.* **10**, 557–560. 10.1016/s0968-0896(01)00313-3 11814842

[bb11] Sheldrick, G. M. (2008). *Acta Cryst.* A**64**, 112–122. 10.1107/S0108767307043930 18156677

[bb12] Spek, A. L. (2009). *Acta Cryst.* D**65**, 148–155. 10.1107/S090744490804362X PMC2631630 19171970

